# A Chip for Estrogen Receptor Action: Detection of Biomarkers Released by MCF-7 Cells through Estrogenic and Anti-Estrogenic Effects

**DOI:** 10.3390/s17081760

**Published:** 2017-08-01

**Authors:** Konstanze Gier, Claudia Preininger, Ursula Sauer

**Affiliations:** Center for Health & Bioresources, AIT Austrian Institute of Technology GmbH, Konrad Lorenz Str 24, 3430 Tulln, Austria; konstanze.gier@ait.ac.at (K.G.); claudia.preininger@ait.ac.at (C.P.)

**Keywords:** endocrine-disrupting chemicals, protein microarray, bisphenol A, cell proliferation

## Abstract

The fluorescence-based multi-analyte chip platform for the analysis of estrogenic and anti-estrogenic substances is a new in vitro tool for the high throughput screening of environmental samples. In contrast to existing tools, the chip investigates the complex action of xenoestrogens in a human cell model by characterizing protein expression. It allows for the quantification of 10 proteins secreted by MCF-7 cells, representing various biological and pathological endpoints of endocrine action and distinguishing between estrogen- and anti-estrogen-dependent secretion of proteins. Distinct protein secretion patterns of the cancer cell line after exposure to known estrogen receptor agonists ß-estradiol, bisphenol A, genistein, and nonylphenol as well as antagonists fulvestrant and tamoxifen demonstrate the potential of the chip. Stimulation of cells with Interleukin-1ß shifts concentrations of low abundant biomarkers towards the working range of the chip. In the non-stimulated cell culture, Matrix Metalloproteinase 9 (MMP-9) and Vascular Endothelial Growth Factor (VEGF) show differences upon treatment with antagonists and agonists of the estrogen receptor. In stimulated MCF-7 cells challenged with receptor agonists secretion of Monocyte Chemoattractant Protein (MCP-1), Interleukin-6 (IL-6), Rantes, and Interleukin-8 (IL-8) significantly decreases. In parallel, the proliferating effect of endocrine-disrupting substances in MCF-7 cells is assessed in a proliferation assay based on resazurin. Using ethanol as a solvent for test substances increases the background of proliferation and secretion experiments, while using dimethyl sulfoxide (DMSO) does not show any adverse effects. The role of the selected biomarkers in different physiological processes such as cell development, reproduction, cancer, and metabolic syndrome makes the chip an excellent tool for either indicating endocrine-disrupting effects in food and environmental samples, or for screening the effect of xenoestrogens on a cellular and molecular level.

## 1. Introduction

The World Health Organization defines endocrine-disrupting chemicals (EDCs) as “exogenous substances or mixtures that alter functions of the endocrine system and consequently cause adverse health effects in an intact organism, or its progeny, or (sub) populations” [1]. EDCs originate from natural sources, as for instance phytoestrogens from soy bean, or from anthropogenic sources, e.g., food packaging materials such as bisphenol A, pesticide residues in food such as atrazine, or environmental pollutants such as alkylphenols [2,3,4,5,6]. A screening of several endocrine active substances showed that they interact with different nuclear receptors such as estrogen receptors, androgen receptor, or peroxisome proliferative receptors. EDCs are able to inhibit or activate these receptors and to disrupt the normal endocrine function by altering circulating hormone levels. They act together with endogen hormones and show low dose, additive, and synergistic effects [7,8,9,10]. Since EDCs interfere with the endocrine system of an organism, their actions may result in symptoms of metabolic syndrome, reproductive dysfunction, and cancer [11].

Thus, there is a strong need for test procedures characterizing estrogenic and anti-estrogenic action of chemicals and their involvement in biological processes. Existing in vitro platforms examine the interaction with or activation of nuclear receptors (e.g., Calux^®^ assay, YES/YAS yeast based, ELRA) [12,13,14], the hormone responsive cell proliferation (e.g., E-Screen) [15], gene products and gene activity (e.g., DNA microarrays) [16], the secretion of single biomarkers (e.g., Vitellogenin) [17], or the enzyme activity within the steroidogenesis [18]. The hormone-like activity of substances is often evaluated with several methods in parallel in order to minimize the chance of false positive and especially false negative results [19]. The strong cell wall of yeast, for instance, can prevent the penetration of lipophilic substances to the target location and cause false negative results of the YES test. Moreover, the transferability of yeast data for the risk assessment for different species, especially humans, is questionable. Commonly used cell-based receptor assays such as Calux^®^ show an interference of estrogenic and anti-estrogenic activity, which complicates substance-dependent assignment to the effect itself [20]. In addition, there are no methods characterizing the cell proteome after exposure to EDCs [19]. Therefore, we aimed to develop an in vitro tool that is capable of a) high throughput screening of environmental samples for endocrine-disrupting effects in a human cell model and b) providing information on their complex cellular effects at the protein level.

**Figure 1:**
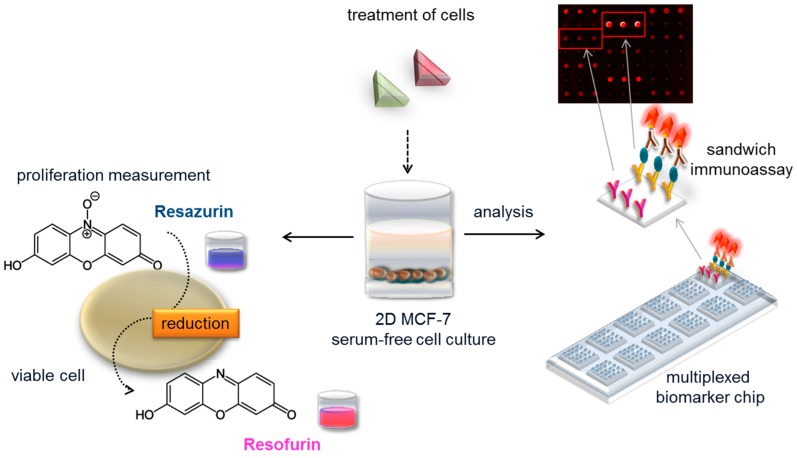
Experimental setup of the biomarker chip and proliferation platform. MCF-7 cells in serum-free cell culture are exposed to e.g., food samples containing estrogen receptor agonists and antagonists; biomarkers released are quantified in supernatant with a multiplexed protein microarray based on fluorescence detection. The on-chip sandwich immunoassay employs immobilized antibodies to capture the biomarker and a fluorescently labeled detection antibody for read out. In parallel, the proliferative effect in hormone-sensitive cancer cell line MCF-7 is measured with a resazurin assay.

Biomarker panels are used in medical diagnostics where single markers cannot achieve the required accuracy [21]. Multiparameter panels are especially meaningful for fast and highly specific diagnosis [22,23,24], disease and targeted therapy monitoring, as well as patient stratification [25], because they draw a picture of the metabolism of individuals. Furthermore, biomarker panels can be used to detect exposure to xenobiotics and the related metabolic processes, both on a cell and on an organism level. In light of this, we developed a fluorescence-based multiplexed protein microarray showing the complex action of estrogenic and anti-estrogenic substances based on human MCF-7 cell culture and a parallel proliferation measurement (see Figure 1).

The supernatant of serum-free MCF-7 cell culture, exposed to estrogen receptor agonists and antagonists present in environmental samples, is used for biomarker detection with the protein microarray. In parallel, the proliferative effect of test substances on hormone-sensitive cancer cell line MCF-7 is assessed with a resazurin proliferation assay. The microarray shows secretion patterns of 10 biomarkers after exposure to estrogen receptor agonists and antagonists, and provides indications for their involvement in: estrogen receptor interaction, steroid synthesis, cancer, metabolic syndrome, and reproductive and developmental processes. The tool overcomes penetration problems by using a human cancer cell line and avoids the interference of different effects by measuring the protein expression of whole cells as an endpoint after exposure to EDCs in comparison to well-known agonists and antagonists of the estrogen receptor.

## 2. Materials and Methods

### 2.1. Cell Line and Cultivation

The hormone-sensitive breast cancer cell line MCF-7 was obtained from American Type Culture Collection (ATCC, Manassas, VA, USA). The cells were cultivated routinely in Dulbecco´s Modified Eagle´s Medium (DMEM) (Sigma Aldrich, St. Louis, MO, USA) supplemented with 10% heat-inactivated fetal bovine serum (FBS), 1% non-essential amino acid (NEAA), 2 mM L-glutamine and penicillin/streptomycin (Thermo Fischer Scientific, Waltham, MA, USA) at 37 °C, 5% CO_2_ and a humidity of 95% in cell culture flasks (Falcon^®^ Corning Inc., Corning, NY, USA). Cells were grown for five days to 80% confluence and then trypsinated by a 10% dilution of a 0.5% trypsin-ethylenediaminetetraacetic acid (EDTA) mixture (Thermo Fischer Scientific, Waltham, MA, USA) in phosphate buffered saline (PBS) without magnesium and calcium (Sigma Aldrich, St. Louis, MO, USA).

### 2.2. Cell Cultivation in Serum-/Phenol Red-Free Medium

For biomarker secretion and proliferation assays, cells were cultured in serum-free medium which was introduced by the following adaption phase. MCF-7 cells were seeded at a density of 5 × 10^4^ cells per well in Costar^®^ cell culture plates, 96-well, flat bottom (Corning Incorporated, Corning, NY, USA) in 200 µL DMEM supplemented with 10% FBS, 1% NEAA, 2 mM L-glutamine and pen/step for one day at 37 °C, 95% humidity, and 5% CO_2_. Cells were allowed to attach on the well surface for 24 h before changing to DMEM/F-12 Gibco™. Dulbecco’s Modified Eagle Medium/Nutrient Mixture F-12 (DMEM/F-12) with Hepes, L-glutamine was supplemented with pen/strep, bovine serum albumin (BSA) solution, and insulin/transferrin/selenium (ITS) mixture (Thermo Fischer Scientific, Waltham, MA, USA) but without phenol red. After 24 h, the medium was removed and DMEM/F-12 without insulin was added to increase the proliferative effect, as well as to allow the acclimatization of cells in the changed environment. Transferrin (Thermo Fischer Scientific, Waltham, MA), sodium selenite (Sigma-Aldrich, St. Louis, MO), BSA solution, and pen/strep were added. The cell layers were washed with 300 µL PBS (-Ca/-Mg) before changing the medium.

### 2.3. Cell Treatment for Biomarker and Proliferation Assays

After 72 h, the cells were treated with 1 nM ß-estradiol, 1 nM tamoxifen, 10 nM fulvestrant, 1 µM nonylphenol, 1 µM bisphenol A (BPA), and 1 µM genistein (Sigma-Aldrich, St. Louis, MO, USA) with and without stimulation by 10 ng/mL human recombinant IL-1ß (eBioscience, San Diego, CA, USA) in 200 µL DMEM/F-12 without insulin for 48 h. After 48 h of treatment, 200 µL of each sample was transferred to a 96-well plate and centrifuged for 1 min at 0.3 (×1000) rpm to get rid of cell particles. The samples were stored on ice while preparing the sampling step for the protein microarray. Test substances were dissolved in dimethyl sulfoxide (DMSO) (Sigma-Aldrich, St. Louis, MO, USA) or 99.9% ethanol absolute (ETOH) (Emsure^®^ Merck Millipore, Darmstadt, Germany). The final solvent concentration in the cell culture medium was 0.1%. A sterile stock concentration of 100 mM for each test substance was made and diluted to the desired concentrations with the solvents. The different dilutions were stored in dark glass jars at 4 °C or −20 °C for genistein. After supernatant removal, the cells were washed with 300 µL PBS (-Mg/-Ca) for the resazurin proliferation assay.

### 2.4. Resazurin Proliferation Assay

For this assay, 1 × 10^4^ cells (in the linear range of the standard curve) were seeded per well to determine the proliferation of the cells in microtiter plates with 200 µL of medium. After acclimatization, cells were treated with estradiol, tamoxifen, fulvestrant, and three different EDCs. After 48 h of exposure, the medium was aspirated and the cells were washed with 300 µL of PBS (-Mg/-Ca). 200 µL of fresh medium containing 2 mM Resazurin (Sigma-Aldrich, St. Louis, MO, USA) were added to the wells and incubated for 5 h. No-cell controls were included. The plates were wrapped in aluminium foil and incubated for 4 h in a cell incubator at 37 °C, 5% CO_2_, 95% humidity. Afterwards, 150 µL aliquots of each sample and controls were transferred into white fluorescence measurement plates, namely Falcon™ White Opaque 96-Well Tissue Culture Plates (Corning, Corning, NY, USA). Fluorescence signals were read at λex = 560 nm and λem = 590 nm on a plate reader.

### 2.5. Materials and Reagents for the Protein Microarray

The proprietary ARChip Epoxy [26] was used as a protein microarray immobilization platform. Anti-human IL-6 (MQ2-13A5), recombinant IL-6 protein, biotinylated anti-human IL-6 (MQ2-39C3), as well as anti-human MCP-1 (5D3-F7), recombinant MCP-1, biotinylated anti-human MCP-1 (2H5), and recombinant IL-1ß for the stimulation of the cells were purchased from eBioscience (San Diego, CA, USA). Anti-human IL-8 (H8A5), anti-human CXCL10 (IP-10) (J036G3), anti-human Rantes (J047C5), recombinant protein IL-8, recombinant protein CXCL10 (IP-10), recombinant protein Rantes, and the biotinylated anti-human IL-8 (E8N1), biotinylated anti-human CXCL10 (IP-10) (Poly5194), biotinylated anti-human Rantes (Poly5197) were obtained from Biolegend (San Diego, CA, USA). Human IGFBP-3 Antibody (E8N1), Recombinant Human IGFBP-3, Biotinylated Antihuman IGFBP-3, Antibody Human IGF-I, Mouse IgG1 (MAb56408), Recombinant Human IGF-I, CF Recombinant Human IL-11, Human IGF-I Biotinylated Affinity Purified PAb, Goat IgG, Monoclonal Anti-human IL-11 Antibody, Biotinylated Anti-human IL-11 (22616), Anti hVEGF 165 (26503), Recombinant Human VEGF 165, VEGF Antibody anti-hVEGF biotinylated, Human Anti-hMMP-9 (36020), Recombinant Human MMP-9, and Biotinylated Anti-human MMP-9 Antibody were obtained from R&D Systems (Minneapolis, MN, USA). Labelled streptavidin Dy647 was from Dyomics (Jena, Germany). Polysorbate 20 (Tween 20), sodium deoxycholate, sodium chloride (NaCl), calcium chloride (CaCl) were purchased from Sigma (St. Louis, MO, USA). Phosphate buffered saline (PBS, pH 7.2, 10X) was from Thermo Fischer Scientific (Waltham, MA, USA) and Tris (hydroxymethyl) aminomethane (Tris) was from AMRESCO (Cleveland, OH, USA).

### 2.6. Protein Microarray Fabrication, Processing, and Scanning

The probes were diluted for spotting in sterile 1× PBS (pH 7.2)/0.01% sodium deoxycholate to concentrations of 0.4 mg/mL for IL-6, IL-8, IL-11, CXCL10, Rantes, MCP-1, IGF-1, IGFBP-3, MMP-9, and 0.5 mg/mL for VEGF. The capture antibodies were arrayed in triplicates onto ARChip Epoxy with an Arrayit Nanoprint^TM^ contact spotter (Arrayit corporation, Sunnyvale, CA, USA). Twelve identical arrays were spotted on each slide with a SMP3 pin at a relative humidity of 50%. The spot-to-spot distance was 350 μm. To achieve immobilization of the probes, slides were stored at 4 °C for at least three days. The appropriate spotting conditions such as composition of print buffer, humidity during spotting, and antibody concentration were established previously [27]. The blocking was performed in 1× PBS (pH 7.2)/0.1% Tween 20 for half an hour. After washing two times in 1× PBS (pH 7.2), the slides were dried with compressed air and mounted into the hybridization cassette (Arrayit Corporation, Sunnyvale, CA, USA) holding four slides and generating 4 × 12 separated arrays. For setting up calibration curves with at least 10 standards for the quantitative assays, a dilution series of mixtures of all analytes in DMEM F12-BSA (minus insulin) was prepared. For an abbreviated procedure, only two to three calibration standards in the linear range and the zero standards were used. Nine replicates for each calibration point (three arrays with three replicate spots) were made, which were distributed on different array fields on three slides. The standards and the cell culture samples (six biological each with three technical replicates) were incubated for 2.5 h. After washing three times with 1× PBS (pH 7.2)/0.1% Tween-20, slides were incubated for 45 min with 50 μL of biotinylated antibody mixtures in buffer J (100 mM TRIS, 100 mM NaCl, 10 mM CaCl_2_ and 0.1% Tween 20; pH 7.4) with final concentrations of 1 μg/mL each. Succeeding another washing cycle, 2 µg/mL Dy647 streptavidin (in buffer J) was added. Slides were incubated for 45 min and washed two times with PBS-T, two times with 1× PBS, then dried with compressed air and stored in the dark until scanning. All incubation steps were carried out at room temperature on a rotary shaker. The slides were measured at λex = 635 nm, λem = 670 nm at optimal photomultiplier tube (PMTs) voltages for each analyte with a confocal laser scanner (LS100, Tecan Group Ltd., Männedorf, Switzerland).

The cross reactivity of each single biomarker with all non-specific capture antibodies of the panel was analyzed and calculated as a ratio of unspecific to specific fluorescence signal in percent of concentration well within the linear range.

### 2.7. Statistical Data Analysis

Spot segmentation and data analysis of the arrays were made using GenePix^®^ Pro 7.0 software (Molecular Devices, LLC Sunnyvale, CA, USA). Background corrected mean signals were calculated from nine technical and six biological replicates for the cell culture samples. Data points that were out of mean signal values ± standard deviation (SD) were excluded. Standard curves were set up with OriginPro 8G using a four-parameter fit; bar graphs with Graph Pad Prism 5.0. Data were plotted with mean ± standard error of mean (SEM). The limit of detection (LOD) was calculated as the mean zero concentration plus three SDs for the limit of quantification (LOQ), plus 10 SDs of the blank of the measured fluorescence intensities [28]. Recovery rates were calculated displaying the percent-relation between calculated concentrations and expected concentrations. The proliferation values were calculated by averaging the fluorescence signals of the non-cell control and subtracting these values from each sample value. The fluorescent values of the samples were averaged and plotted as means with SEM. Normal distribution of the data was tested with the Kolmogorov-Smirnov test. The data (stimulation experiment, proliferation) were log-transformed (Y = Log(Y)) or baseline-corrected (treatment-dependent secretion experiments) to the medium control (C0), and statistical analyses were made using a one-way ANOVA and Bonferroni multiple comparison test for alpha = 0.05.

## 3. Results

### 3.1. Compilation of the Biomarker Panel

Based on the literature, we made an initial selection of 26 potentially interesting biomarkers with known estrogen-dependent secretion, representing clues for reproductive and developmental toxicity or links to cancer and metabolic syndrome (for instance: amphiregulin; ET-1; GCDFP-24 (PBCP); GCDFP-15 (PIP); MMP-2; PGE2; pS2 protein; SCF; TGF-ß; IL-18; Platelet-Derived Growth Factor (PDGF); EGF; endostatin; IGFBP-4). Markers such as endostatin, PDGF, and human estrogen-induced protein pS2 had to be excluded, as antibodies were either not commercially available or did not show activity in our assays. Ten biomarkers entered the final panel, including the cytokines Interleukin-6 (IL-6), IL-8, and IL-11, Vascular Endothelial Growth Factor (VEGF), Matrix Metalloproteinase-9 (MMP-9), Monocyte Chemotactic Protein-1 (MCP-1), Chemokine (C-C motif) Ligand 5 (CCL5, Rantes), C-X-C Motif Chemokine 10 (CXCL10, IP-10), Insulin-like Growth Factor-1 (IGF-1), and Insulin-like Growth Factor Binding Protein (IGFBP)-3.

### 3.2. Assay Performance in Serum-Free Cell Culture Medium

For the resulting panel of 10 biomarkers (IL-11, IL-6, IL-8, MCP-1, IGF-1, IGFBP-3, CXCL10, Rantes, MMP-9, VEGF), a multiplexed protein chip was developed, evaluating and optimizing the performance of antibody pairs, protein standards, and assay reagents of the sandwich immunoassay (for calibration curves, see Figure S1 in supplementary data). The sensitivity and specificity of assays processed in buffer were compared to those transferred to the standard medium of MCF-7 cell culture, DMEM supplemented with 10% FBS, and serum-free/phenol-red-free medium DMEM F-12. Sensitivity as expressed by the limit of detection was as good or even better in the standard cell culture medium compared to the assay buffer. The LOD of IL-6, for instance, changed from 18 pg/mL in buffer to 9 pg/mL in DMEM/ 10% FBS, whereas MCP-1 and IGF-1 showed minimal impairment of the detection limit from 7 pg/mL and 115 pg/mL in buffer to 14 pg/mL and 125 pg/mL in DMEM/ 10% FBS, respectively. Interestingly, processing the biomarker arrays in serum-free cell culture medium revealed severe losses in sensitivity for IL-6, IL-11, MCP-1, Rantes, and CXCL10. The signal intensities for IL-11 decreased by around 50% from 48,000 to 25,000 arbitrary units A.U., and the LOD increased from 224 pg/mL to 3653 pg/mL compared to DMEM/ 10% FBS. In addition, cross-reactivity of VEGF antibodies, primarily against IGF-1 (58%), occurred. Upon adding BSA solution in a final concentration of 10 mg/mL to the serum-free medium, we detected an up to 10-fold increase in sensitivity for IL-11, Rantes, CXCL10, and IL-6. In addition, signal intensities for IL-11 and Rantes were improved by 25 to 36% for the highest standard concentration in the assay. The cross-reactivity seen for the biomarker VEGF to IGF-1 decreased by 77%. Also, the recovery rates of IL-6, IL-11, Rantes, VEGF, IGF-1, and MMP-9 were improved (see Table 1). The system recovered the analytes within a range of 80–144%, depending on the biomarker measured, for relevant concentrations within the working range of the assay. Typical working ranges of commercial ELISA kits for single biomarkers are reported in supplementary Table S1.

### 3.3. Quantification of Biomarker Secretion in Cell Culture Supernatant

**Figure 2:**
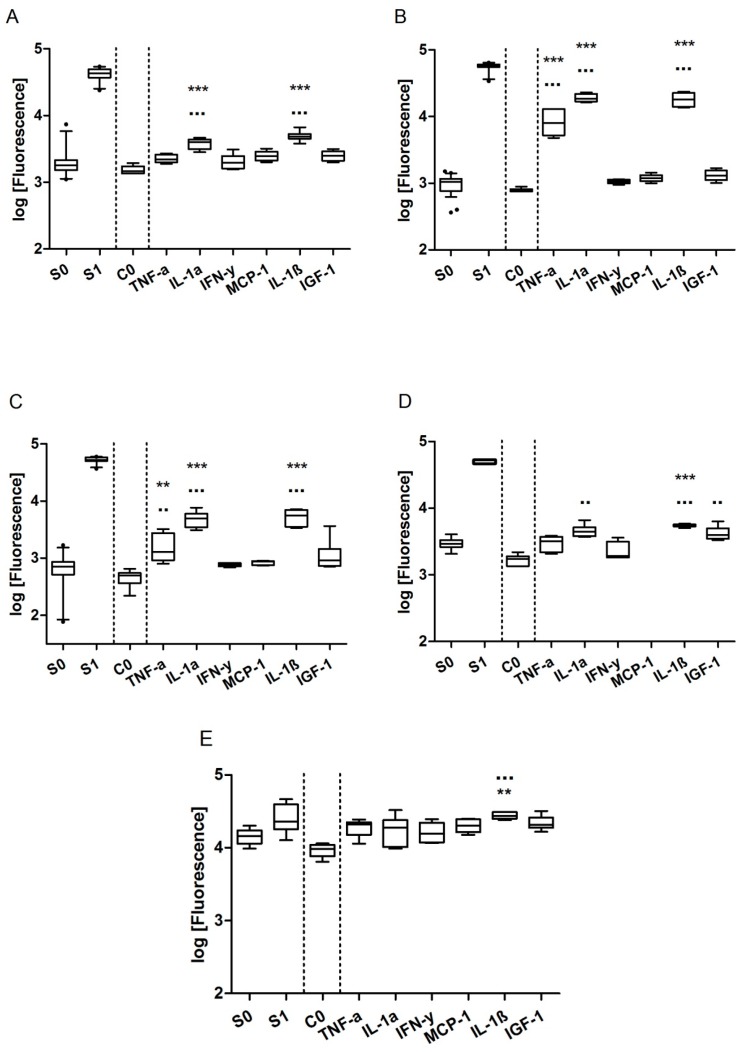
Secretion of IL-8 (**A**), Rantes (B), IL-6 (**C**), MCP-1 (**D**), and CXCL10 (**E**) after stimulation with IGF-1, MCP-1, IL-1a, IL-1ß, TNF-a, and IFN-y. Log of the fluorescence intensities arbitrary units (A.U.) of two standards (without cells) S0 and S1 and the medium control (with cells) C0 without stimulation as well as stimulated cells. Box plots are shown with 5th and 95th quartiles. Significance was tested for stimulation (*n* = 6) against the zero standard S0 (*) and the medium control C0 (▪) for alpha = 0.05 using a one-way-ANOVA and Bonferroni multiple comparison post-hoc test (p ≤ 0.05 (*, ▪), p ≤ 0.01 (**, ▪▪), p ≤ 0.001 (***, ▪▪▪)).

For a successful quantification, the working range of the chip (see Table 1 and Figure S1 in supplementary data) needs to cover the biomarker concentrations secreted by MCF-7 cells. MMP-9, VEGF, IGFBP-3, IL-8, IL-11, IGF-1, and Rantes could be detected with sufficient sensitivity with the chip, albeit IL-8 and Rantes were secreted only in the low pg/mL range and hence difficult to quantify. The secretion of CXCL10, MCP-1, and IL-6 was below the LOD of their respective standard curves. Since it is known that MCF-7 cells are able to increase the secretion of proteins by stimulation with cytokines or growth factors, we tested various stimulants in order to shift the secretion of biomarkers to higher concentration levels, better fitting the sensitivity of the chip. The concentrations considered for stimulation were deduced from literature and from specifications on the biological activity provided by the supplier [29,30,31,32]. After separate stimulation of MCF-7 cells with either 10 ng/mL TNF-α, 50 ng/mL IL-1α, 10 ng/mL IL-1β, 50 ng/mL IGF-1, 10 ng/mL IFN-γ, or 50 ng/mL MCP-1 for 24 to 48 h, all biomarkers were secreted at levels matching the measuring range of the chip. In Figure 2, the secretion of low abundant markers is shown without (C0) and with stimulation. Fresh medium without cells and devoid of proteins of the panel was used as a zero standard (S0), medium spiked with a 7.7 ng/mL (a concentration in the linear range of the standard curves) biomarker standard was used as a positive control (S1), and medium with cells (C0) was used as a control for a successful stimulation. Stimulation with IL-1α, IL-1β, and TNF-α particularly resulted in up to one log higher expression of the analytes compared to the other stimulants and no stimulation. IL-1ß stimulation yielded especially high signals, notably for IL-6, IL-8, Rantes, MCP-1, and CXCL10, markers which could not be detected or which were only detected in low quantities without stimulation. Consequently, for the experimental setup, in parallel to untreated MCF-7 cells, stimulation with IL-1ß and accumulation of the secretion products over 48 h was chosen. In the following experiments, we could quantify nine of the 10 biomarkers at concentrations ≥50 pg/mL after 48 h of cell culture stimulation. Only IGF-1 secretion fell below the detection limit upon treatment with receptor agonists.

### 3.4. Effect of Solvents on Biomarker Secretion and Cell Proliferation

Hormones such as estradiol and estrogen-like substances are hydrophobic compounds and hence require an organic solvent rather than water or cell medium. Clearly, it has to be tested if the vehicles in which the substances are dissolved have an influence on the outcome and interpretation of cell experiments. For the hormones and xenobiotics tested herein, ethanol (EtOH) and DMSO are suitable solvents. We used the evaluation of those two solvents as the first showcase for the utility of the biomarker chip. A proliferation assay was employed as a reference, based on fluorescence detection through the biochemical conversion of resazurin to the fluorescent resofurin.

**Figure 3:**
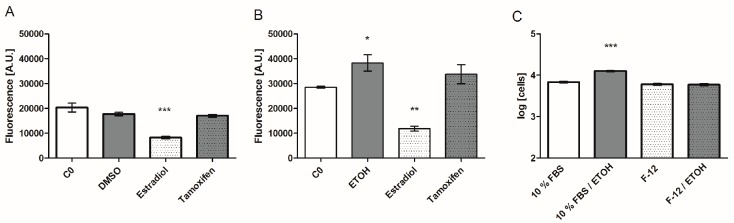
Secretion of the biomarker Rantes in IL-1ß stimulated cells challenged with solvents (**A**) 0.1% DMSO and (**B**) 0.1% EtOH compared to untreated cells (C0), and estrogen receptor agonist (estradiol) and antagonist (tamoxifen) treatment. (**C**) Proliferation of MCF-7 cells in standard medium and serum-free/phenol-red-free medium with and without 0.1% EtOH. Proliferation data are log transformed. Bar graphs are plotted as means ± SEM. Significance was tested (*n* = 5 (**A**,**B**); *n* = 4–8 (**C**)) against the C0 control with a one-way ANOVA and a Bonferroni multiple comparison test for alpha = 0.05 (p ≤ 0.5 (*), p ≤ 0.01 (**), p ≤ 0.001 (***)).

Effects of 0.1% EtOH and 0.1% DMSO on MCF-7 proliferation and biomarker expression were tested, in both DMEM/ 10% FBS and DMEM F-12. The secretion of biomarkers VEGF, Rantes, IL-6, and IGFBP-3 increased after treatment with 0.1% EtOH, suggesting direct stimulation of their expression, while neither MMP-9 expression nor the other markers were affected. The strongest upregulation was seen for Rantes, of about 34% compared to the medium control (see Figure 3B). Also, cell proliferation in serum-supplemented cell culture with 0.1% EtOH compared to the medium control increased, while the serum-free cell cultures were not significantly affected. Experiments with 0.1% DMSO, a concentration also reported in the literature as not cytotoxic [33], showed no significant effect on the proliferation and secretion compared to the medium control after 48 h, and was used in the following experiments for dissolving test substances.

### 3.5. Specific Biomarker Secretion Patterns and Proliferative Effect of ER Agonists and Antagonists

**Figure 4:**
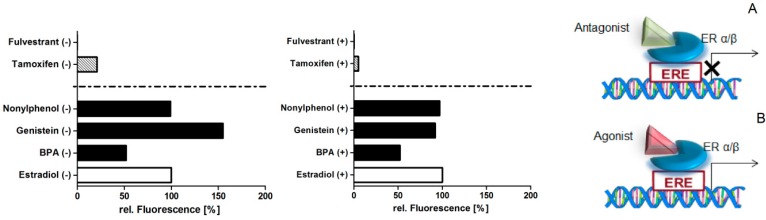
Proliferative effect of MCF-7 cells after treatment with the agonists estradiol, bisphenol A (BPA), genistein, and nonylphenol as well as the antagonists tamoxifen and fulvestrant of the estrogen receptor—left graph without (−) and right graph with IL-1ß stimulation (+). The binding of agonists (**B**) to the estrogen receptor results in the proliferation of cells, while the antagonistic (**A**) action inhibits the proliferation. Data (*n* = 6) are baseline-corrected to the estradiol treatment and the medium control, and plotted as relative fluorescence.

MCF-7 cells were exposed to 1 µM nonylphenol, bisphenol A, and genistein, known estrogen receptor agonists, for 48 h. As positive controls for estrogenic action, 1 nM 17ß-estradiol, the most affine endogen ligand of the estrogen receptor, was employed; for anti-estrogenic action, 1 nM tamoxifen (in breast tissue) and 10 nM fulvestrant were employed. These concentrations were chosen according to the maximal response they showed in proliferative assays without being cytotoxic [15,34,35,36]. Nuclear receptors act as transcription factors and are switched on and off by agonistic and antagonistic ligands, respectively (Figure 4A,B). An increase in proliferation after treatment with estrogen receptor agonists, particularly agonists to the receptor isoform ERα, was expected compared to the antagonists. With and without IL-1ß stimulation, nonylphenol showed the same proliferative effect as estradiol, bisphenol A reduced it by half, while genistein evoked 50% more response without stimulation (Figure 4).

**Figure 5:**
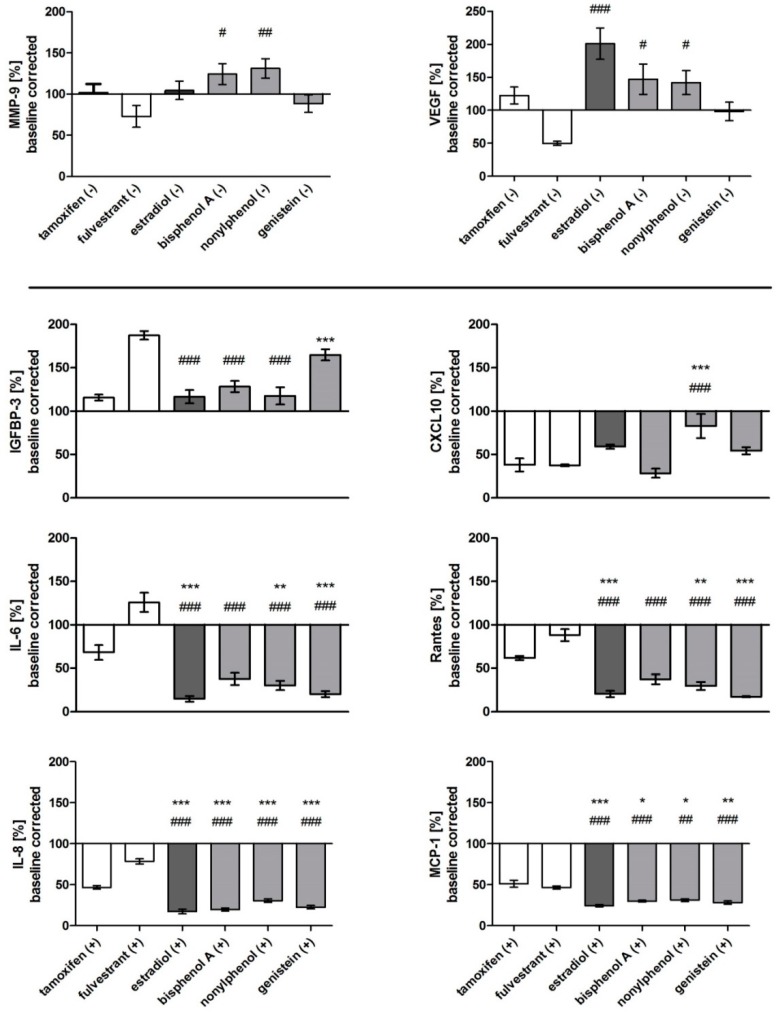
Relative secretion of MMP-9, VEGF, IGFBP-3, CXCL10, IL-6, Rantes, IL-8, and MCP-1 after exposure to estrogen receptor agonists (estradiol, BPA, nonylphenol, genistein) and antagonists (fulvestrant, tamoxifen) without (-) or with (+) stimulation by IL-1ß for 48 h. Data are baseline-corrected and plotted as means ± SEM in percent to the C0 control. Significance against tamoxifen (*) and fulvestrant (#) was tested for *n* = 6 using a one-way-ANOVA and a Bonferroni multiple comparison post-hoc test for alpha = 0.05 (p ≤ 0.05 (*, #), p ≤ 0.01 (**, ##), p ≤ 0.001 (***, ###)).

Figure 5 depicts the biomarkers which resulted in a specific secretion upon exposure to tested estrogens and anti-estrogens. Without stimulation, VEGF and MMP-9 exhibited significant differences in cells challenged with fulvestrant compared to estrogen receptor agonists, however, no differential response to agonists and antagonists with stimulation was found. Other biomarkers also showed distinct expression patterns upon treatment with agonists and antagonists when cells were grown in the presence of IL-1ß. The relative expression of IL-6, IL-8, Rantes, and MCP-1 was significantly lower for estradiol, nonylphenol, and genistein compared to tamoxifen and fulvestrant; the effect of bisphenol A was less prominent. IGFBP-3 was downregulated after estradiol, bisphenol A, and nonylphenol exposure, while genistein treatment resulted in a similar secretion to fulvestrant after IL-1ß stimulation. Next to IGFBP-3, only MMP-9 and IL-11 were upregulated compared to the medium control when challenged with endocrine active substances, while the other biomarkers were expressed in lower levels. Table 2 summarizes these results, showing the relative expression of markers translated into a color code.

## 4. Discussion

The fluorescence-based multi-analyte chip platform for the analysis of estrogenic and anti-estrogenic substances is a new in vitro tool for the high throughput screening of environmental samples. In contrast to existing tools, the chip investigates the complex action of xenoestrogens in a human cell model by characterizing protein expression. It allows for the quantification of 10 proteins secreted by MCF-7 cells, representing various biological and pathological endpoints of endocrine action and distinguishing between estrogen- and anti-estrogen-dependent secretion of proteins.

Biomarkers IL-6 and IL-8, VEGF, MMP-9, MCP-1, IL-11, IGF-1, and Rantes are associated with different stages of cancer. MCP-1 and IL-11 are stimulators within the development and progression of breast cancer, while IL-6 takes part in survival, proliferation, and cell migration processes [37,38,39,40]. IL-8 as well as VEGF are involved in tumor angiogenesis and metastasis in breast cancer, while MMP-9 is related to the degradation of the extracellular matrix and tumor cell invasion [41,42,43,44]. Co-expression of Rantes, the main inducer of cell invasion, and MCP-1 is related to advanced stages of cancer [38]. In contrast, CXCL10 and IGFBP-3 were shown to have anti-malignant properties by anti-angiogenic action or the inhibition of breast cancer cell proliferation [45,46,47]. IGF-1 has a main role in cell proliferation and development, and is additionally involved in the pathology of adipositas, diabetes mellitus, and hypertriglyceridemia, factors of the metabolic syndrome; its secretion is stimulated via growth hormones like estradiol [48,49]. IGFBP-3 is the main IGF transport protein and its expression correlates with the estrogen receptor status [50,51]. The biomarker IL-6 is able to stimulate aromatase secretion and thereby estrogen biosynthesis. In addition, elevated expression was seen for IL-6 in adipose tissue in connection to obesity and insulin resistance [48]. In contrast, the multifunctional cytokine IL-11 acts as an effective inhibitor of adipogenesis, one of the factors of the metabolic syndrome [52]. Next to IGF-1, MCP-1, IL-8, VEGF, MMP-9, Rantes, IL-6, and IGFBP-3 are up- or downregulated after treatment with estrogen or estrogen-like substances [41,42,43,53,54,55,56]. MCP-1, Rantes, IGF-1, and IL-8 are additionally involved in developmental or reproductive processes such as menstruation, pregnancy, and endometriosis [57,58]. The role of the selected biomarkers in different physiological processes such as cell development, reproduction, cancer, and metabolic syndrome makes the chip an excellent tool for either indicating endocrine-disrupting effects in food and environmental samples or for screening new xenoestrogens and their additive or synergistic effects on a cellular and molecular level. Further, the microarray can easily be extended with additional markers, provided that reagents of adequate quality become available.

Multiplexed on-chip sandwich immunoassays with fluorescent read-out were chosen because the highly parallel, miniaturized format of the chip outperforms conventional bioanalytical techniques in screening applications. Apart from antibody quality, interferences from the sample matrix are likely to affect the sensitivity, specificity, and reproducibility of immunoassays. In our biomarker chip, no sample preparation step of cell supernatants is intended in order to keep the experimental setup simple and the protein expression unbiased. Due to the generally low abundance of the targeted proteins, dilution of the sample is not an option. The drop in assay performance upon changing the matrix from DMEM/ 10% FBS to DMEM F-12 can be explained by the absence of serum proteins from fetal serum in the latter. Serum albumins such as BSA or human serum albumin (HAS), as part of the natural environment of antibodies, are known to minimize or even prevent non-specific binding, to increase signal intensities, to ensure specificity, and to stabilize the biomolecules immobilized on the surface [59,60]. Nevertheless, for markers IL-6, IL-8, Rantes, MCP-1, and CXCL10, known to be secreted in vitro in low quantities by MCF-7 cells [29,32,39,45,54], the sensitivity of the chip was not sufficient for a reliable detection in the low pg/mL range (see Table 1). Stimulation of MCF-7 cells with cytokines and growth factors, most effectively with interleukin-1ß, mimicking biological processes such as inflammation, promoted secretion to measurable levels for very low abundant biomarkers (Figure 2). There is evidence suggesting that IL-1ß in combination with genistein and fulvestrant showed additive effects for IL-11, IGFBP-3, and MMP-9, and that treatment with BPA and IL-1ß even resulted in synergistic effects, probably due to positive feedback loops within the cells. Further experiments would be needed to verify whether there are additive or synergistic effects of IL-1ß and the estrogen receptor agonists and antagonists.

The proliferation of MCF-7 cells is regulated via hormones. Therefore, they are used in validated toxicological methods such as the E-Screen to detect estrogen-like activity of xenobiotics compared to estradiol via proliferation measurements [15]. Hormone-regulated proliferation of MCF-7 cells evaluated by means of fluorescence measurements using the conversion of resazurin to resofurin was therefore used as a reference for our chip experiments. We found more prominent differences in proliferation without IL-1ß stimulation, especially with genistein. Increased proliferation upon challenging cells with genistein was also found in Reference [61]. Bisphenol A showed only half of the effect compared to estradiol. Such differences in proliferation were also detected with the E-Screen developed by Soto et al. [15]. They described nonylphenol with a relative proliferative effect of 100% as a full agonist of the estrogen receptor, while Bisphenol A showed only 85% [62]. Antagonists fulvestrant and tamoxifen were below 30% compared to estradiol, but still showed a proliferative response. After stimulation with IL-1ß, not only the proliferative effect of genistein was decreased to the level of estradiol, but tamoxifen also showed a drop in proliferation (Figure 4).

Both tools, the proliferation assay and the chip, were employed to test the effects of organic solvents, which may be a necessary vehicle to introduce the test substances in the cell culture medium. Ethanol was reported to be less cytotoxic compared to DMSO, which showed low cytotoxicity in concentrations above 0.5% [33]. Nevertheless, we detected an increase of cell proliferation in serum-supplemented cell culture with 0.1% EtOH compared to the medium control, but not in the serum-free and phenol-red-free medium (see Figure 3C). A possible explanation might be the additive effect of weak estrogenic phenol-red and ethanol. Our results are supported by Etique et al. [63], showing an enhanced proliferation of MCF-7 cells especially in the presence of low concentrations of EtOH. EtOH was reported to directly affect the expression of MMP-9, especially at concentrations ≥0.3% [64], and it significantly stimulated the ER alpha activity in a dose-dependent manner [65]. However, we did not detect any effects on the secretion of MMP-9 related to 0.1% EtOH, but on VEGF, Rantes, IL-6, and IGFBP-3. Based on the literature and our own data, we concluded that the experimental background error by EtOH is higher compared to DMSO at low concentrations, and that ≤0.1% DMSO should be the preferred solvent for sample preparation. The system was validated by exposing MCF-7 cells to well-known estrogen-like endocrine disruptors, resulting in obviously related secretion patterns (Table 2) and proliferation as estradiol-treated cells. The biomarker patterns resulting from treatments with the antagonist fulvestrant and with estradiol were clearly distinctive. Anti-estrogens can have agonistic as well as antagonistic effects on MCF-7 cell proliferation in estrogen-lacking conditions. Tamoxifen in particular was mentioned to show agonistic effects in low concentrations [66]. The biomarker expression pattern of tamoxifen stands out compared to that of fulvestrant-treated cells and supports this finding.

IL-6, IL-8, MCP-1, and Rantes showed significantly downregulated expression for all estrogen active substances compared to the full antagonist fulvestrant, while the other biomarkers featured a more diverse secretion (Figure 5 and Table 2). Genistein is able to induce the downregulation of the ERα mRNA and protein levels, which would explain the similar secretion levels of MMP-9, VEGF, and IGFBP-3 following fulvestrant treatment [67]. The selective estrogen receptor antagonist fulvestrant accelerates the degradation of the estrogen receptor as well [68]. Furthermore, it is reported that the phytoestrogen genistein can bind to both isoforms of the estrogen receptor ERα and ERβ with a high affinity, which also could explain differences in secretion and proliferation.

Additional screening of estrogen receptor agonists and antagonists with graded binding affinities, in varied concentrations and treatments, for different time periods, will be necessary to refine the specific secretion patterns and gain a comprehensive and differentiated understanding of EDCs mode of action. Further, the chip can be employed to elucidate protein secretion within disease development or involvement in processes such as steroid synthesis. An extension of the system offers various options, which can further promote the biomarker chip as new tool for the detection and characterization of endocrine action. Such extensions may include adding more biomarkers; integrating other human cell lines; applying 3D cell culture in order to be closer to in vivo conditions; co-cultures with e.g., fibroblasts; or the use of primary cells.

## Figures and Tables

**Table 1:** Limit of detection (LOD), limit of quantification (LOQ), half maximal effective concentration (EC50), recovery rates, and coefficient of variation (CV) of sandwich assays for IL-6, IL-8, IL-11, MCP-1, CXCL10, Rantes, IGF-1, VEGF, MMP-9, and IGFBP-3 on the multiplexed chip platform in Dulbecco’s Modified Eagle Medium/Nutrient Mixture F-12 DMEM/F-12 cell culture medium supplemented with 10 mg/mL bovine serum albumin BSA. Data of LOD, LOQ, and EC50 are shown as means ± SEM of three experiments. The coefficient of variation is calculated as the mean of eleven standards of three experiments. The recovery rate is given for a spiked concentration within the linear range of the curves.

Biomarker	LOD pg/mL	LOQ pg/mL	EC50 pg/mL	Recovery Rate %	CV %
MCP-1 (CCL2)	4 ± 2	16 ± 7	2431± 1668	83	14
IL-6	5 ± 2	16 ± 6	3916 ± 1155	132	13
Rantes (CCL5)	16 ± 7	46 ± 21	4400 ± 1841	144	11
VEGF	20 ± 12	61 ± 25	3923 ± 938	131	15
IL-8	53 ± 17	196 ± 97	3216 ± 678	80	22
CXCL10 (IP-10)	91 ± 24	321 ± 119	7852 ± 2275	116	19
IL-11	157 ± 26	530 ± 199	26,330 ± 13,022	105	13
IGFBP-3	581 ± 254	1630 ± 477	30,644 ± 3011	104	17
MMP-9	669 ± 197	2035 ± 447	49,071 ± 7070	118	19
IGF-1	803 ± 122	2553 ± 453	39,523 ± 10,874	87	15

**Table 2:** Color-coded biomarker secretion patterns. MCF-7 cells (stimulated: +, non-stimulated: -) challenged with tamoxifen, fulvestrant, estradiol, genistein, bisphenol A, and nonylphenol, and marker expression quantified with the chip relative to untreated cells (%). Colors are graded in steps of 20%.

	Tamoxifen		Fulvestrant		Estradiol		Genistein		BPA		Nonylphenol
MMP-9 -	102		73		105		89		124		131
VEGF -	122		50		201		98		147		142
											
IGFBP-3 +	133		213		130		190		145		136
IL-6 +	68		126		15		20		38		30
IL-8 +	47		78		17		23		20		31
MCP-1 +	51		47		24		28		30		31
Rantes +	62		88		21		17		37		30
CXCL-10 +	38		38		59		54		28		83
IL-11 +	139		328		192		268		295		198
IGF-1 +	n.a.		n.a.		n.a.		n.a.		n.a.		n.a.

Colour code: 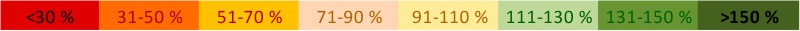.

## Supplementary Materials

The following are available online at http://www.mdpi.com/1424-8220/17/8/1760/s1. Figure S1: Standard curves of CXCL10, IGF-1, IL-6, VEGF, IL-8, Rantes, IL-11, MCP-1, IGFBP-3, and MMP-9 generated with 11 standards using serum free cell culture medium as a matrix. Table S1. Typical working ranges of standard ELISA kits for single biomarkers in microtiter plate format.
