# Supplementary material for: A Chip for Estrogen Receptor Action: Detection of Biomarkers Released by MCF-7 Cells through Estrogenic and Anti-Estrogenic Effects
Source: Sensors (Basel). 2017 Aug 1;17(8):1760. doi: 10.3390/s17081760 (PMC5579769; doi:10.3390/s17081760)
Supplement: Supplementary file 1 [file sensors-17-01760-s001.pdf]

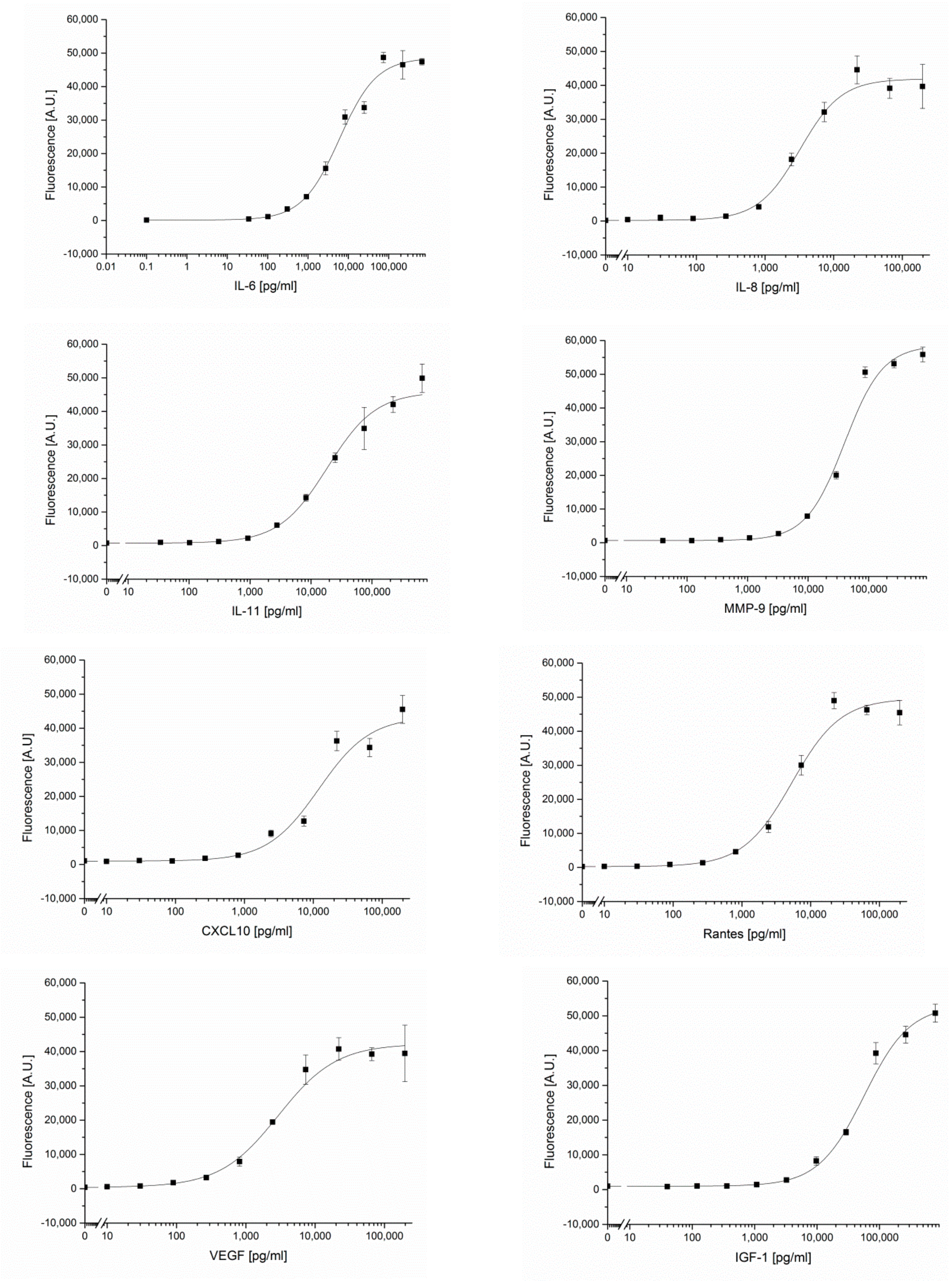

Figure S1. Standard curves of IL-6, IL-8, IL-11, MMP-9, CXCL10, Rantes, VEGF, MCP-1, IGFBP-3, and IGF-1 generated with 11 standards using serum-free cell culture medium as a matrix. Background-corrected mean signals were calculated from nine technical replicates and plotted as means ± SD. Curves were fitted using a four-parametric model.

Table S1. Typical working ranges of standard ELISA kits for single biomarkers in microtiter plate format.

Biomarker	Range Comm. ELISA [pg/mL]	Supplier
MCP-1 (CCL2)	15.6–1000	eBioscience, San Diego, USA
IL-6	3.1–200	eBioscience, San Diego, USA
Rantes (CCL5)	31.2–2000	RnD Systems, Minneapolis, USA
VEGF	15.6–1000	RnD Systems, Minneapolis, USA
IL-8	31.2–2000	Biolegend, San Diego, USA
CXCL10 (IP-10)	15.6–1000	Biolegend, San Diego, USA
IL-11	15.6–1000	RnD Systems, Minneapolis, USA
IGFBP-3	800–50,000	RnD Systems, Minneapolis, USA
MMP-9	300–20,000	RnD Systems, Minneapolis, USA
IGF-1	100–6000	RnD Systems, Minneapolis, USA
